# Inhibition by curcumin of multiple sites of the transforming growth factor-beta1 signalling pathway ameliorates the progression of liver fibrosis induced by carbon tetrachloride in rats

**DOI:** 10.1186/1472-6882-12-156

**Published:** 2012-09-16

**Authors:** Qun-yan Yao, Bei-li Xu, Ji-yao Wang, Hong-chun Liu, Shun-cai Zhang, Chuan-tao Tu

**Affiliations:** 1 Department of Gastroenterology and Hepatology, Zhongshan hospital, Fudan University, 180# Fenglin Road, Shanghai, 200032, People’s Republic of China

**Keywords:** Curcumin, Hepatic stellate cells, Liver fibrosis, Transforming growth factor-beta, Smads, Connective tissue growth factor

## Abstract

**Background:**

At present there is no effective and accepted therapy for hepatic fibrosis. Transforming growth factor (TGF)-β1 signaling pathway contributes greatly to hepatic fibrosis. Reducing TGF-β synthesis or inhibiting components of its complex signaling pathway represent important therapeutic targets. The aim of the study was to investigate the effect of curcumin on liver fibrosis and whether curcumin attenuates the TGF-β1 signaling pathway.

**Methods:**

Sprague–Dawley rat was induced liver fibrosis by carbon tetrachloride (CCl_4_) for six weeks together with or without curcumin, and hepatic histopathology and collagen content were employed to quantify liver necro-inflammation and fibrosis. Moreover, the mRNA and protein expression levels of TGF-β1, Smad2, phosphorylated Smad2, Smad3, Smad7 and connective tissue growth factor (CTGF) were determined by quantitative real time-PCR, Western blot, or immunohistochemistry.

**Results:**

Rats treated with curcumin improved liver necro-inflammation, and reduced liver fibrosis in association with decreased α-smooth muscle actin expression, and decreased collagen deposition. Furthermore, curcumin significantly attenuated expressions of TGFβ1, Smad2, phosphorylated Smad2, Smad3, and CTGF and induced expression of the Smad7.

**Conclusions:**

Curcumin significantly attenuated the severity of CCl_4_-induced liver inflammation and fibrosis through inhibition of TGF-β1/Smad signalling pathway and CTGF expression. These data suggest that curcumin might be an effective antifibrotic drug in the prevention of liver disease progression.

## Background

Liver fibrosis and its end stage, cirrhosis, represent the final common pathways of virtually all chronic liver diseases [1]. Chronic damage to the liver from, for example, hepatitis virus infection or alcohol abuse has a negative impact on the wound healing response of the liver, with excessive deposition of extracellular matrix (ECM) leading to organ fibrosis and increased loss of liver function [1,2]. Hepatic stellate cells (HSCs) are the primary effector cells in liver fibrosis, orchestrating the deposition of ECM in normal and fibrotic liver [3,4]. Cytokine-mediated activation of HSCs into a myofibroblast-like phenotype is a key event during liver fibrogenesis [2,4,5].

Current evidence suggests that the process of hepatic fibrosis is driven by a complex network of cytokines, foremost being transforming growth factor (TGF)-β1 and connective tissue growth factor (CTGF) [1-3]. Following liver damage, parenchymal cells (hepatocytes) and mesenchymal cells (Kupffer cells, endothelial cells, pit cells, HSCs) release cytokines, which participate in HSC activation and ECM synthesis [4,5]. High concentrations of TGF-β1 are often found in patients with hepatic fibrosis, with TGF-β1 implicated as a mediator of fibrosis in many liver diseases. Release of TGF-β1 by necrotic hepatocytes may be one of the first signals to activate adjacent quiescent HSCs, resulting in their transdifferentiation into proliferative, fibrogenic and contractile myofibroblasts. Therefore, TGF-β1 has been regarded as the master cytokine in liver fibrogenesis [2,3,6].

TGF-β1 acts through different signalling pathways, the most important being the canonical Smad pathway [6,7]. Smad proteins are divided into three functional classes: receptor-regulated (Smads 1, 2, 3, 5, and 8), common mediator (Co-Smad4), and inhibitor (Smads 6 and 7) [6,7]. TGF-β signals through transmembrane receptors that stimulate cytoplasmic Smad proteins, which modulate the transcription of target genes, including those encoding ECM proteins, such as procollagen-I and -III [8]. Disruption of the TGF-β/Smad signalling, mainly due to loss of Smad3 or increased Smad7 expression, has been found to result in resistance to tissue fibrosis in many organs [3,6-8], suggesting that reducing TGF-β synthesis or inhibiting components of its complex signalling pathways represent important therapeutic targets [2,3,6,9].

CTGF, also known as CCN family 2 (CCN2), is induced by TGF-β and is considered a downstream mediator of the effects of TGF-β on fibroblasts [2,10]. The CTGF gene promoter contains a functional SMAD binding site, and TGF-β induction of CTGF in dermal fibroblasts is R-Smad-dependent [11]. CTGF acts synergistically with TGF-β1 to promote matrix protein deposition and fibrogenesis, both *in vitro* and *in vivo*[12]. Subcutaneous or intraperitoneal injection of either TGF-β or CTGF/CCN2 individually does not induce persistent fibrosis, whereas co-injection of both proteins together results in sustained fibrosis [12,13]. CTGF is overexpressed in human liver fibrosis/cirrhosis of various aetiologies and in experimental liver fibrogenesis [12-16]. It is a major fibrogenic signal, with TGF-β-dependent activity when produced in hepatocytes, and TGF-β-independent activity when derived from HSCs [17]. Furthermore, studies in various animal models have demonstrated that targeting genes encoding CTGF/CCN2 proteins in liver fibrosis may be therapeutically beneficial [2,12,15,18]. Knock-down of CTGF by injection of small interfering RNA (siRNA) into the portal veins of rat livers prevented carbon tetrachloride (CCl_4_)-induced fibrosis by inhibiting TGF-β induction and HSC activation [18]. Therefore, CTGF/CCN2 may be an important target for experimental trials of anti-fibrotic agents [12,15,18].

Curcumin, a polyphenol (diferuloylmethane), is the main active compound found in the perennial plant *Curcuma longa* (commonly known as turmeric) [19]. Curcumin has various types of biological activities, including anticancer, antiviral, antioxidant, and anti-inflammatory properties [19-22]. Moreover, several recent reports showed that curcumin has beneficial effects in animal models of liver injury and cirrhosis [19,23-25]. However, its mechanism of action in liver injury remains to be determined, and identifying novel biological activities of curcumin is very important preclinically and clinically. We have therefore evaluated the ability of curcumin to prevent the development and progression of carbon tetrachloride (CCl_4_) -induced liver fibrosis in rats, and we investigated the molecular mechanisms underlying the effects of curcumin in this model.

## Methods

### Materials

Carbon tetrachloride (CCl_4_) and curcumin were purchased from Sigma Chemical Co., Ltd. (St. Louis, MO) and dissolved in olive oil and PBS, respectively, for experiments in animals. Antibodies included human polyclonal anti-TGF-β1 antibody (Promega, Madison, WI), rabbit anti-TGF-β1 polyclonal antibody (Boster Co., Ltd., Wuhan, China), goat polyclonal anti-CTGF antibody (Santa Cruz Biotechnology, Santa Cruz, CA), rabbit monoclonal anti-Smad2, anti-Smad3 and anti-Smad7 antibodies (Epitomics, Burlingame, CA), rabbit polyclonal anti-phospho-Smad2 (*Ser465/467*) antibody (Cell Signaling Technology, Danvers, MA), rabbit polyclonal anti-collagen I antibody (Abcam, Cambridge, MA), rabbit polyclonal α-SMA antibody (Dako Diagnostic, San Antonio, TX), mouse monoclonal anti-GADPH antibody (Kangcheng Biotech Co., Ltd., Shanghai, China), and HRP-conjugated goat anti-rabbit and goat anti-mouse immunoglobulin G (Beijing CowinBioscience Co., Ltd., Beijing, China).

### Animals and experimental protocols

Male Sprague–Dawley rats weighing 180–220 g were obtained from the animal centre of Fudan University (Shanghai, China) and maintained in an environmentally controlled room (23 ± 2°C, 55 ± 10% humidity) with a 12-hour light/dark cycle and free access to food and water. Liver fibrosis was induced by administration of 2 ml of CCl_4_/olive oil (1:1, v/v)/kg body weight by intraperitoneal (i.p.) injection twice weekly for up to 6 weeks. Fifty rats were randomly divided into four groups. Rats in group 1 (n = 10) received twice weekly injections of olive oil (vehicle control). Rats in group 2 (n = 10) were injected with olive oil, as in group 1, and received oral curcumin (200 mg/kg) [22-24]. Rats in group 3 (n = 15) received twice weekly injections of CCl_4_ plus oral curcumin, and rats in group 4 (n = 15) were injected with CCl_4_ group and received oral PBS. The study was performed in accordance with the guiding principles for the care and use of laboratory animals approved by the Research Ethics Committee of Zhongshan Hospital, Fudan University (No. 2011–87).

### Histopathological evaluation

Harvested liver tissues were fixed in 10% neutral buffered formalin and embedded in paraffin. Slices 4 μm thick were prepared and stained with haematoxylin and eosin and Sirius red according to standard procedures. Liver necroinflammatory activity was evaluated semiquantitatively according to the METAVIR scoring system [26], with 0 = none, 1 = mild, 2 = moderate, and 3 = severe. Fibrosis was also evaluated semiquantitatively, with red-stained fibrotic areas of sections stained with Sirius red measured on a video screen display using a digital image analyser (400 × magnification, KS400, Carl Zeiss Vision, Germany) by a technician blinded to the treatment regimen. Ten fields were randomly selected from each section; and six to eight rats in each group were examined. Results were expressed as the percentage of fibrotic area in each field [27].

### Immunohistochemical evaluation

Liver tissue sections were dewaxed, hydrated and subjected to heat-induced antigen retrieval. Sections were blocked and incubated overnight at 4°C with (1) rabbit anti-TGF-β1 polyclonal antibody (1:50), (2) anti-Smad2 antibody (1:50), (3) anti-Smad3 antibody (1:100), (4) anti-CTGF antibody (1:100) and (5) anti-α-SMA antibody (1:100), with all antibodies diluted in TBS–2% bovine serum albumin. Negative-control antibodies were species-matched and, where appropriate, immunoglobulin G subclass-matched Ig fractions. The sections were washed and incubated with secondary antibodies. Colour was developed by incubation for 5–10 min with 3, 3′-diaminobenzidine tetrachloride, and specific staining was visualized by light microscopy.

### Western blot analysis

Liver samples were homogenized and centrifuged at 10,000 *g* at 4°C for 10 minutes. The protein concentrations of the supernatants were determined using the BCA protein colourimetric assay kit (Pierce Biotechnology, Rockford, IL), with bovine serum albumin as the standard. Protein samples (50 μg) were separated by SDS-PAGE on 10-12% gradient gels and transferred onto polyvinylidene difluoride membranes. The membranes were incubated in blocking buffer (5% nonfat milk powder in TBST [100 mM Tris–HCl, pH 7.5, 0.9% NaCl, 0.1% Tween 20]) for 3 h, followed by incubation overnight at 4°C with gentle shaking with specific primary antibodies against CTGF (1:500), Smad2 (1:500), phospho-Smad2 (1:500), Smad7 (1:1000), anti-TGF-β1 (1:500), and α-SMA (1:1000), Collagen-I (1:500) and with monoclonal anti-GAPDH antibody (1:5000) as a loading control. After washing off the unbound antibody with TBST, the expression of antibody linked protein was determined using ECL™ Western Blotting Detection Reagents (Amersham Pharmacia Biotech Inc., Piscataway, NJ). The optical density of the bands was measured and quantified by Image J (National Institutes of Health, Bethesda, MD) and expressed in arbitrary units.

### Real-time quantitative RT-PCR

Total RNA was extracted from frozen liver tissues using Trizol reagent according to the manufacturer’s protocol (Life Technologies, Grand Island, NY). RNA extracts were reverse-transcribed with random hexamers and avian myeloblastosis virus reverse transcriptase using a commercial kit (Perfect Real Time, SYBR® PrimeScriP™ TaKaRa, Shiga, Japan). Real-time RT-PCR for quantitative assessment of mRNA expression was performed on an ABI Prism 7000 Sequence Detection system (Applied Biosystems, Tokyo, Japan) according to the manufacturer’s protocol. Probes and primers for TGF-β1, CTGF, Smad2, Smad3, Smad7, and GAPDH (Table 1) were purchased from Applied Biosystems. The level of expression of each target gene was normalized relative to the expression of GAPDH mRNA in that sample using the ΔCt method. Relative differences in gene expression among groups were determined using the comparative Ct (ΔΔCt) method and fold expression was calculated as 2^−ΔΔCt^, where ΔΔCt represents ΔCt values normalized relative to the mean ΔCt of control samples.

**Table 1:** Primer sequences used in this study

**Target gene**	**Forward primer (5'-3')**	**Reverse primer (5'-3')**
TGFβ1	GTGGCTGAACCAAGGAGACG	CAGGTGTTGAGCCCTTTCCAG
CTGF	GCCCTAGCTGCCTACCGACT	ACAGGCTTGGCAATTTTAGGC
Smad2	TCGGAAAGGGCTGCCACAT	AGTCATCCAGAGGCGGCAG
Smad3	AGGGCTTTGAGGCTGTCTACC	ACCCGATCCCTTTACTCCCA
Smad7	GGGGGAACGAATTATCTGGC	CGCCATCCACTTCCCTTGT
GAPDH	AGTGCCAGCCTCGTCTCATAG	CGTTGAACTTGCCGTGGGTAG

### Statistical analysis

All statistical analyses were performed using SPSS statistical software (SPSS for windows, version 15.0). All data are presented as mean ± SD. Differences in measured parameters among groups were analysed by one-way analysis of variance followed by the Tukey test when F was significant. Differences were considered significant at p values less than 0.05.

## Results

### Curcumin administration ameliorates hepatic injury, inflammation and fibrosis in liver tissue of CCl_4_ treated rats

**Figure 1:**
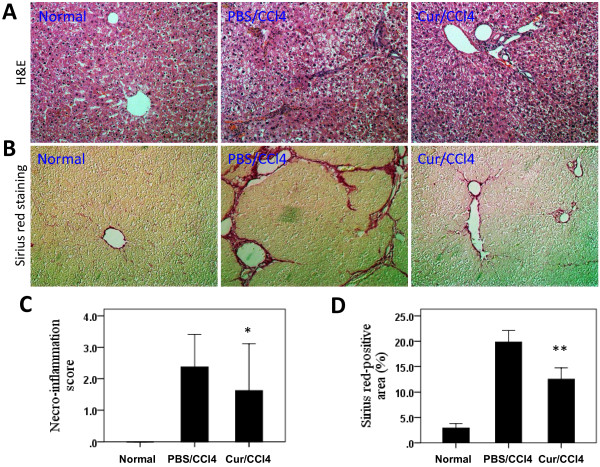
**Curcumin (Cur) administration ameliorates hepatic inflammation and fibrosis induced by CCl**_**4**_**administration.** (**A**) Haematoxylin and eosin (H&E) staining of the livers (original magnification, ×200). (**B**) Evaluation of liver fibrosis by Sirius red staining of liver sections (original magnification, ×200). (**C**) Semiquantitative evaluation of liver necro-inflammatory activity according to the METAVIR scoring system. *P < 0.05 for Cur/CCl_4_ vs. PBS/CCl_4_ (n = 8/group). (**D**) Quantitative image analysis of hepatic Sirius red staining using a computerized image analysis system; **P < 0.001 for Cur/CCl_4_ vs. PBS/CCl_4_ (n = 6-8/group).

No rats died in group1 and group2 during the 6 weeks of experimental period, while 4 and 5 rats died in group 3 (Curcumin/CCl_4_) and group 4 (PBS/CCl_4_), respectively. The ability of curcumin to protect rat livers against injury and fibrogenesis induced by CCl_4_ was evaluated histologically (Figure 1A and 1B). Rats injected with CCl_4_ alone, in the absence of oral curcumin, developed severe liver injury and fibrosis, as evidenced by the prominent steatosis of hepatocytes, pericellular and periportal bridging fibrosis, and distortion of liver architecture. However, oral administration of curcumin daily for 6 weeks significantly attenuated alterations in liver histology. Normal rats treated with curcumin exhibited normal histological changes, similar to those in normal, untreated control rats (data not shown).

Prolonged CCl_4_ treatment induced the formation of necrotic areas in rat livers, with considerable infiltration of inflammatory cells into areas surrounding the centrilobular veins (Figure 1A). Treatment with curcumin, however, attenuated the severity of inflammation and necrosis induced by CCl_4_ (Figure 1A) as well as significantly reducing necroinflammation scores in these animals (Figure 1C).

### Curcumin inhibits hepatic fibrogenesis and activation of hepatic stellate cells in liver tissue of CCl_4_ treated rats

To assess the impact of curcumin on CCl_4_-induced hepatic fibrogenesis, liver sections were stained with Sirius red to detect the deposition of collagens. Rats receiving CCl_4_ for 6 weeks developed significant fibrosis, with initial stages showing the characteristic patterns of perivenular and periportal deposition of connective tissue, along with the development of thin portal-to-portal septa and slight evidence of architectural distortion resulting in micronodular fibrosis (Figure 1B). Few areas containing healthy hepatocytes were present and collagen deposition with septa bridging portal regions was detected. However, CCl_4_-treated rats orally administered 200 mg/kg per day curcumin displayed thinner septa and more preserved hepatic parenchyma. Furthermore, collagen deposition in the livers of CCl_4_-treated rats was confirmed by computerized image analysis of the fibrotic area, whereas curcumin treatment significantly prevented the progression of CCl_4_-induced fibrosis (12.50% vs. 19.85%, *P* < 0.001; Figure 1D).

**Figure 2:**
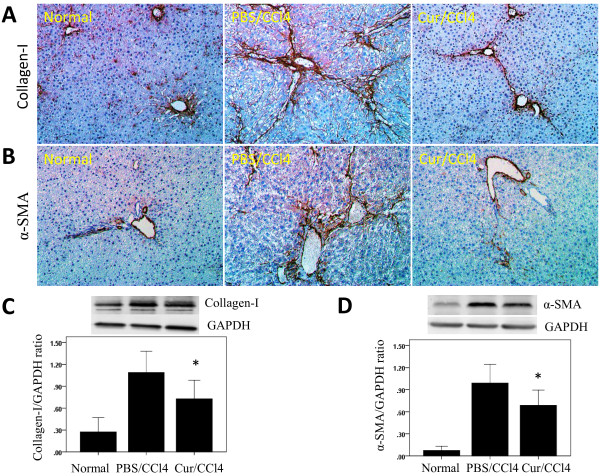
**Effects of curcumin on activation of hepatic stellate cells (HSCs) and collagen type I protein expression in the liver.** (**A**, **B**) Immunohistochemical staining of type I collagen and alpha-smooth muscle actin (α-SMA) in the liver (original magnification, ×200). (**C**, **D**) Western blotting analysis of hepatic collagen-I and α-SMA expression. Liver tissue was lysed and subjected to western blot analysis for collagen-I and α-SMA, and the loading control GAPDH. Blots obtained from several experiments were analysed using densitometry, and the densitometric values were pooled from four animals per group and shown as means ± SD in the bar graph. *P < 0.01 for Cur/CCl_4_ vs. PBS/CCl_4_.

HSC proliferation has been reported augmented when these cells were cultured on type I collagen, the predominant matrix component increased in liver fibrosis. We therefore analysed the effect of curcumin on the expression of type I collagen protein. We found that, compared with control rats, CCl_4_-treatment resulted in marked collagen-I accumulation in the liver, with increased collagen-I expression in the periportal areas and areas of bridging fibrosis in the liver, as shown by immunohistochemistry (Figure 2A). In contrast, rats administered curcumin showed significantly reduced collagen-I deposition (Figure 2A). These findings were supported by western blotting analysis. Although collagen-I was detected in all groups of rats, its highest intensity was in rats receiving CCl_4_ alone (Figure 2C). However, administration of curcumin to CCl_4_ injected rats significantly decreased the levels of collagen-I protein by 33.3%.

Activated HSCs, which expression myogenic markers such as α-SMA, are considered central ECM-producing cells within the injured liver [1,28], playing a significant role in collagen-I deposition. We sought to determine whether curcumin treatment inhibited the activity of HSCs, we immunohistochemically stained tissue sections with antibody to α-SMA. We observed α-SMA in all groups of animals, with the highest intensity in rats injected with CCl_4_ alone, with administration of curcumin significantly decreasing the levels of α-SMA expression (Figure 2B). These findings were substantiated by western blotting experiments, which showed that the amount of α-SMA was reduced following curcumin treatment (Figure 2D). These findings indicate that prevention with curcumin efficiently reduces the numbers of activated HSCs in damaged livers.

### Curcumin administration inhibits hepatic TGF-β1 expression in liver tissue from CCl_4_ injected rats

**Figure 3:**
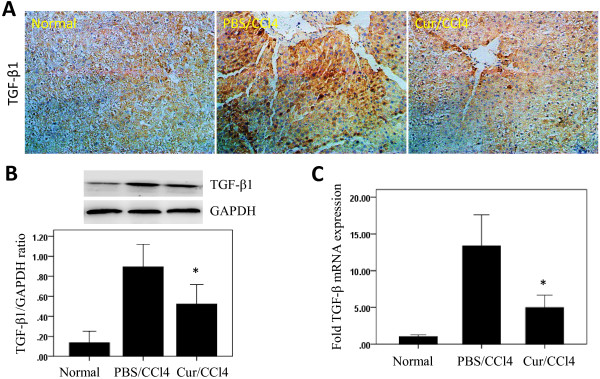
**Effects of curcumin (Cur) on transforming growth factor beta1 (TGF-β1) expression in the liver.** (**A**) Immunohistochemical assessment of TGF-β1 in the liver (original magnification, ×200). (**B**) Western blotting analysis of hepatic TGF-β1 expression. Liver tissue was lysed and subjected to western blot analysis for TGF-β1, and the loading control GAPDH. Blots obtained from several experiments were analysed using densitometry, and the densitometric values were pooled from four animals per group and are shown as means ± SD in the bar graph. *P < 0.05 vs. rats treated with CCl_4_ alone. (**C**) Analysis of hepatic TGF-β1 mRNA expression by quantitative PCR. TGF-β1 mRNA levels were normalized relative to those of GAPDH mRNA in each sample, and values are expressed as the mean ± SD fold increase compared with the normal control group (n=6). *P < 0.01 for Cur/CCl_4_ vs. PBS/CCl_4_.

Since the multifunctional cytokine TGF-β1 has been found to play important roles in the development of liver diseases [6], we measured TGF-β1 expression in the livers of CCl_4_ treated rats. We found that immunoreactivity against TGF-β1 was weak in the livers of normal control rats, but was increased in the hepatic parenchyma of CCl_4_ treated rats, especially in areas around the central veins (Figure 3A). Moderate staining of TGF-β1-positive hepatocytes around the central veins was also observed after curcumin treatment. The levels of expression of TGF-β1 protein were also evaluated by western blotting. TGF-β1 was detected in all groups of rats, with the highest intensity in rats treated with CCl_4_ alone, and significantly decreased levels in CCl_4_ injected animals treated with curcumin (Figure 3B).

Using quantitative real-time PCR, we assayed the intrahepatic expression of TGF-β1 mRNA 6 weeks after CCl_4_ injection, with or without curcumin treatment. We found that CCl_4_ markedly increased intrahepatic TGF-β1 mRNA levels, but this increase was attenuated in rats that received oral curcumin (Figure 3C).

### Curcumin induces Smad7 and inhibits Smad2, P-Smad2 and Smad3 expression in liver tissue of CCl_4_ treated rats

TGF-β1 signals are mediated by Smad. Following ligand binding to TGF-β type II receptors (TβRII), TGF-β type I receptors (TβRI) are activated and bind Smad2 and Smad3 proteins. These proteins are subsequently phosphorylated, forming an oligomeric complex with Smad4 [2,3]. Smad7 is an inhibitor of TGF-β1 expressed in response to prolonged TGF-β1 signalling; it binds to TβRI and abrogates the effects of TGF-β1 [2,3]. We therefore analysed the effects of CCl_4_ and curcumin on the expression of the TGF-β1 downstream signalling molecules, phosphorylated Smad2 (P-Smad2), total Smad2, Smad3, and Smad7 in the liver.

**Figure 4:**
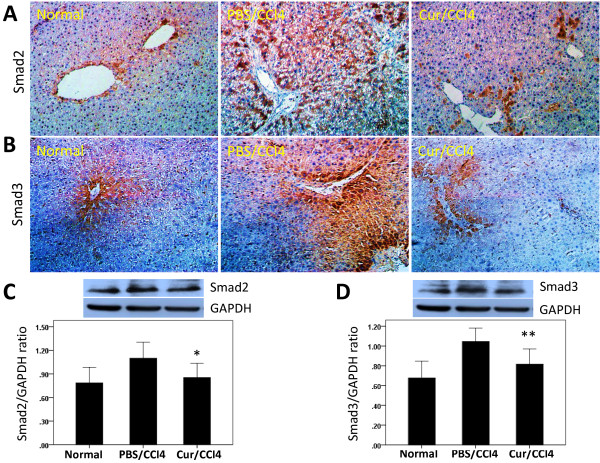
**Effects of curcumin (Cur) on the expression of Smad2 and Smad3 proteins in rat liver tissue.** (**A**, **B**) Immunohistochemical staining for Smad2 and Smad3 in the liver (original magnification, ×200). (**C**, **D**) Western blot analysis of hepatic Smad2 and Smad3 expression. Liver tissue was lysed and subjected to western blot analysis for Smad2 and Smad3 and the loading control GAPDH. Blots obtained from several experiments were analysed using densitometry, and the densitometric values were pooled from four animals per group and shown as means ± SD in the bar graph. *P < 0.05 for Cur/CCl_4_ vs. PBS/CCl_4_, **P < 0.01 for Cur/CCl_4_ vs. PBS/CCl_4_.

We found that Smad2 and Smad3 were expressed in the cytoplasm, but not the nuclei, of liver cells in all groups of rats (Figure 4A, 4B). In control rats, a few hepatocytes were weakly positive for Smad2 and Smad3, whereas after CCl_4_ treatment, strong positivity was observed, mainly around the central veins and scattered throughout the lobules. Moderate staining for Smad2 and Smad3 was also observed in some hepatocytes from rats treated with CCl_4_ and curcumin.

**Figure 5:**
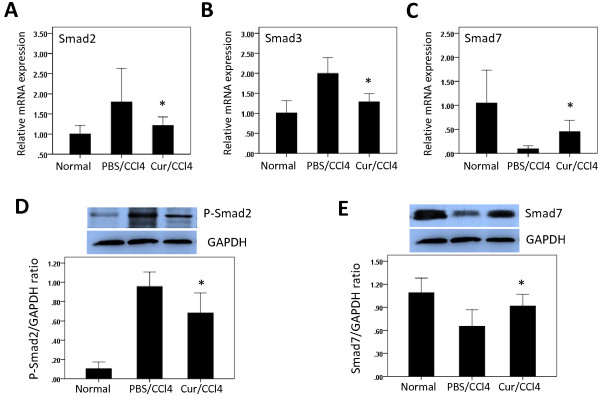
**Curcumin induces Smad7 and inhibits Smad2, P-Smad2, and Smad3 mRNA and protein expression in liver tissue of CCl**_**4**_**injected rats.** (**A**-**C**) The levels of Smad2 (**A**), Smad3 (**B**) and Smad7 (**C**) mRNA in liver tissue were determined by quantitative real-time RT-PCR. Smad2, Smad3 and Smad7 mRNA levels were normalized relative to GAPDH mRNA expression in each sample, and values are expressed as the mean ± SD fold increase over normal control group (n=6). *P < 0.01 for Cur/CCl_4_ vs. PBS/CCl_4_. (**D**-**E**) Western blots analysis of hepatic P-Smad2 and Smad7 expression, with GAPDH as the loading control. Blots obtained from several experiments were analysed using densitometry, and the densitometric values were pooled from four animals per group and are shown as means ± SD in the bar graph. *P < 0.05 for Cur/CCl_4_ vs. PBS/CCl_4_.

Western blotting experiments showed that Smad2 (Figure 4C), P-Smad2 (Figure 5D), and Smad3 (Figure 4D) were expressed in all groups, with the highest intensity of each in rats treated with CCl_4_ alone. Administration of curcumin to CCl_4_-injected rats, however, significantly decreased the levels of these proteins, while increasing the expression of Smad7 protein (Figure 5E).

We also assayed the intrahepatic expression of Smad2, Smad3 and Smad7 mRNAs by quantitative real-time PCR. We found that CCl_4_ administration for 6 weeks markedly enhanced intrahepatic Smad2 and Smad3 mRNA expression, but that this was attenuated in rats also administered curcumin (Figure 5). In contrast, CCl_4_ administration reduced intrahepatic Smad7 mRNA levels, whereas administration of curcumin to CCl_4_-injected rats enhanced Smad7 mRNA (Figure 5C).

### Curcumin inhibits CTGF expression in liver tissue of CCl_4_ treated rats

**Figure 6:**
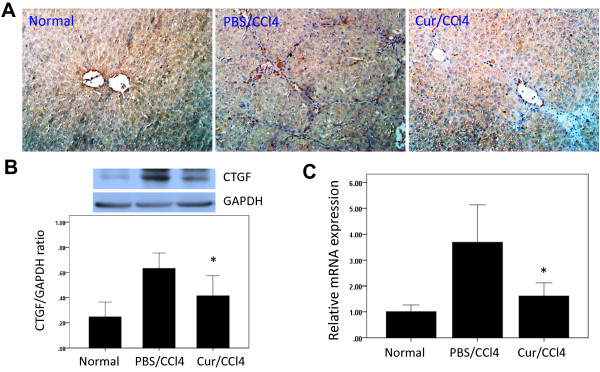
**Curcumin inhibits connective tissue growth factor (CTGF) expression in liver tissue of CCl**_**4**_**treated rats.** (**A**) Immunohistochemical staining for CTGF in rat liver (original magnification, ×200). (**B**) Western blots analysis of hepatic CTGF expression and GAPDH as loading control. Blots obtained from several experiments were analysed using densitometry, and the densitometric values were pooled from four animals per group and are shown as means ± SD in the bar graph. *P < 0.05 for Cur/CCl_4_ vs. PBS/CCl_4_. (**C**) The levels of CTGF mRNA in liver tissue were determined by quantitative real-time RT-PCR and normalized relative to GAPDH mRNA expression in each sample, with values expressed as the mean ± SD fold increase over normal control group (n=6). *P < 0.01 for Cur/CCl_4_ vs. PBS/CCl_4_.

To assess the location and dynamics of CTGF expression in liver fibrosis, we immunohistochemically analysed the expression of CTGF protein in rats treated with CCl_4_ for 6 weeks. We found that CTGF was strongly expressed in these livers, with CTGF localizing along the fibrous septa and in different types of liver cells, including HSCs and hepatocytes (Figure 6A). Treatment of these rats with curcumin, however, reduced CTGF expression in hepatocytes and HSCs significantly, to levels observed in control rats. Similarly, western blotting experiments showed that CTGF was expressed in the livers of all three groups, with the highest intensity in rats treated with CCl_4_ for 6 weeks. However, treatment of the latter with curcumin significantly decreased the expression of CTGF (Figure 6B).

When we assayed intrahepatic CTGF mRNA by quantitative real-time PCR, we found that CCl_4_ enhanced intrahepatic CTGF mRNA levels, whereas curcumin attenuated this increase (Figure 6C).

## Discussion

Liver fibrosis is an outcome of many chronic diseases and often results in cirrhosis. Cirrhosis has been associated with significant and life-threatening complications, including liver failure, portal hypertension and hepatocellular carcinoma, and represents a major cause of morbidity and mortality worldwide [2,5]. Mounting clinical and experimental evidence, however, has demonstrated that even advanced fibrosis and cirrhosis are reversible [1,2,4,5]. Therefore, halting the progression from fibrosis to cirrhosis has been considered an important goal in patients with liver diseases [29].

We have shown here that prevention with curcumin (200 mg/kg) significantly attenuated the progression of hepatic fibrosis in rats induced by CCl_4_ injection. Curcumin not only reduced the severity of liver necro-inflammation, but significantly suppressed the activation of HSCs and reduced collagen accumulation and the expression of type I collagen protein. These changes were accompanied by the down-regulation of TGF-β1/Smad signalling and CTGF expression in the livers of rats with hepatic fibrosis.

Curcumin (diferuloylmethane) is an active phenolic compound extracted from the rhizome of the plant *C. longa* Linn (Family: Zingiberaceae), a perennial herb belonging to the ginger family, and native to tropical Southern and Southeast Asia [22,30,31]. Curcumin has potential utility in the prevention and treatment of various diseases, including respiratory conditions, inflammation, liver disorders, diabetic wounds, cough and certain tumours [19-25,30-33]. Recently, several studies have demonstrated that curcumin attenuated experimental liver fibrosis of various aetiologies, including biliary cirrhosis, by mechanisms that included down-regulation of NFκB and TGF-β and the anti-oxidant properties of the latter [19,22-25]. Further investigations of the mechanism of action of this drug are needed, as are additional basic and clinical studies, before recommending this agent for the treatment of patients with chronic liver disorders.

Fibrosis develops as a result of the sustained wound-healing processes that occur in the liver in response to chronic injuries and inflammation. Chronic and repeated hepatic inflammation has been found to result in hepatic fibrosis and cirrhosis [1-4]. Overexpression of TGF-β1 may play a pivotal role in the progression of fibrosis [1-3]. TGF-β1 expression was found to be markedly elevated after hepatic injury, and TGF-β signalling was required for the activation of HSCs [1-3]. TGF-β has been called the master cytokine in liver fibrogenesis, and TGF-β1 synthesis is one of the primary targets in the development of antifibrotic agents [6,29]. We found that curcumin reduced TGF-β1 expression in the livers of CCl_4_-treated rats, suggesting that TGF-β down-regulation by curcumin may be key mechanisms of its antifibrogenic effects. Similarly, in biliary cirrhosis, curcumin showed antifibrogenic activities associated with the down-regulation of TGF-β [32]. Moreover, this antifibrotic effect of curcumin has been confirmed in a TGF-β-driven model of fibrotic lung and kidney diseases [19,33].

It is well known that inflammation is an important element in the initiation and progression of liver fibrosis [1-4]. Chronic hepatic inflammation is accompanied by the upregulation of proinflammatory and chemotactic mediators, including TGF-β, TNF-α, IL-6, IL-8, and MCP-1, mediators that activate HSCs [1-4]. HSC is the pivotal cell type in the development of liver fibrosis and may be the major source of fibrillar collagens (types I, III and IV) and other matrix proteins that accumulate in chronic liver disease [1]. Following any type of liver injury, quiescent HSCs undergo activation to proliferative, fibrogenic, and contractile myofibroblasts with increased expression of α-SMA, a marker of myofibroblast activation *in vitro* and *in vivo*[1,28,29]. We examined the expression of activated HSCs in liver tissue using α-SMA staining and found that curcumin treatment prevented the activation of HSCs. These findings were substantiated by western blotting analysis; showing α-SMA content was reduced following curcumin treatment. Because TGF-β1 is a powerful activator of HSCs, the curcumin-induced decrease in TGF-β1 expression doubtless contributed to changes in this cell type. Taken together, our findings indicate that curcumin directly interferes with the overall process of HSC activation, reducing the numbers of ECM protein transcripts.

TGF-β signals through transmembrane receptors that stimulate cytoplasmic Smad proteins, which in turn modulate the transcription of target genes, including those encoding ECM components, such as procollagen-I and -III [4,34]; Smad 2 and 3 are stimulatory, whereas Smad 7 is inhibitory and is antagonized by Id1 [2,6,7]. Smad3 has been identified as the primary mediator of fibrogenic responses in HSCs, especially in the induction of collagen expression [16,34-37]. Targeted disruption of Smad3 has been found to confer resistance to the development of dimethylnitrosamine-induced hepatic fibrosis in mice [37]. We found that curcumin inhibited Smad3 expression in the livers of CCl_4_-treated rats, compared with rats treated with CCl_4_ alone. To assess activated TGF-β1 signalling in liver tissue, we assayed total Smad2 and Smad2 phosphorylation, finding that curcumin inhibited both total and phosphorylated Smad2. Curcumin has been shown to block the profibrotic activities of TGFβ on human proximal tubule cells *in vitro*, by down-regulating the Smad signalling pathway, suggesting that curcumin may have effects similar to those of serine/threonine protein phosphatases [38]. Moreover, prolonged curcumin treatment *in vivo* was found to significantly reduce TβRII levels and Smad2/3 phosphorylation in response to added TGF-β [33].

In contrast, Smad7 blocks R-Smad phosphorylation and subsequent downstream events by forming a stable complex with activated TβRI [2,3,34,35]. Inhibitory Smad7 is very efficient in blunting the effects of TGF-β in general and has been used to prevent fibrogenesis in chronic diseases of the liver, kidneys, lungs, and skin [2,3]. For example, adenovirus-mediated overexpression of Smad7 in the liver potently blunted bile duct ligation-induced liver fibrosis and efficiently inhibited intracellular TGF-β signalling [35]. Curcumin is regarded as a cytokine inducing effects antagonistic to those of TGF-β, and has been shown to have antifibrotic activity in a variety of organs [18,19]. Our *in vivo* results showed that curcumin administration to rats with CCl_4_-induced liver fibrosis markedly induced Smad7 mRNA and protein expression as compared with rats treated with CCl_4_ alone. Therefore, blocking TGF-β1 production and Smad-dependent signalling have been shown to be successful therapeutic strategies in experimental models of liver fibrosis [7,8,35].

CTGF is a member of the CCN family of proteins and plays a pivotal role in fibrosis of the lungs, skin, kidneys, and heart by affecting ECM production, cell cycle control, and cell adhesion and migration [2,12-15]. CTGF expression has been reported higher in patients with chronic liver fibrosis, including those with diseases such as chronic hepatitis C, non-alcoholic steatohepatitis, and liver cirrhosis [14,15,28]. Moreover, CTGF gene silencing through siRNA was shown to reduce the activation of HSCs, to prevent the upregulation of CTGF and TGF-β1 gene expression and to inhibit the accumulation of connective tissue proteins in the liver [15,39], indicating that CTGF functions as an important profibrogenic cytokine in the liver. Our findings confirmed that chronic CCl_4_ administration markedly enhanced the intrahepatic expression of CTGF mRNA and protein, and that curcumin significantly reduced these CCl_4_-induced increases. Similarly, curcumin has been shown to suppress CTGF expression in HSCs by inhibiting the activation of ERK and NF-κB [40] and to reduce the extent of cardiac fibrosis *in vivo* by markedly inhibiting CTGF expression [41].

Although the main source of CTGF was thought to be HSCs and fibroblasts, recent reports have revealed that hepatocytes also produce CTGF [17,28,42]. Immunohistochemical staining showed that CTGF was located in bile duct epithelial cells, HSCs, and hepatocytes in liver tissue [15-17,43]. TGF-β1 has been shown to stimulate CTGF expression, mainly in primary hepatocytes, but not in HSCs [17,28,42]; suggesting that other cytokines may drive CTGF production during liver fibrogenesis.

## Conclusions

Our findings demonstrate that early and chronic administration of curcumin effectively protected rat livers from CCl_4_-induced liver injury and fibrogenesis. These beneficial effects of curcumin, delaying the progression of hepatic fibrosis, involved inhibiting the TGFβ1/Smad signalling pathway and decreasing CTGF expression. Our results suggest that curcumin may be useful for prevention of hepatic fibrosis in patients with advanced liver disease.

## Competing interests

The authors declare that they have no competing interests.

## Authors’ contributions

QYY and BLX carried out the study and designed the experiments. QYY, HCL, and CTT contributed reagents, materials, analysis tools, and interpretation of data. QYY, HCL, and BLX contributed animal model. JYW and SCZ analyzed data, helped conceive the study, and participated in its design. CTT conceived and designed experiments and wrote the manuscript. All authors read and approved the final manuscript.

## Pre-publication history

The pre-publication history for this paper can be accessed here:

http://www.biomedcentral.com/1472-6882/12/156/prepub
